# Clarifying Evidence-Based Medicine in Educational and Therapeutic Experiences of Clinical Faculty Members: A Qualitative Study in Iran

**DOI:** 10.5539/gjhs.v7n7p62

**Published:** 2015-03-26

**Authors:** Yahya Safari

**Affiliations:** 1 School of Paramedical Sciences, Kermanshah University of Medical Sciences, Kermanshah Iran

**Keywords:** evidence-based medicine, clinical faculty members, clinical experiences

## Abstract

**Introduction:**

Although evidence-based medicine has been a significant part of recent research efforts to reform the health care system, it requires an assessment of real life community and patient. The present study strives to clarify the concept of evidence-based medicine in educational and therapeutic experiences of clinical faculty members of Kermanshah University of Medical Sciences (2014).

**Materials and Methods:**

It was a qualitative study of phenomenology. The population consists of 12 clinical faculty members of Kermanshah University Medical Sciences. Sampling was carried out using a purposeful method. Sample volume was determined using adequacy of samples’ law. Data gathering occurred through semi-structured interviews. Collaizzi pattern was employed for data interpretation concurrent with data gathering.

**Results:**

interpreting the data, three main themes were extracted. They include: 1. Unawareness and disuse (unaware of the concept, disuse, referral to colleagues, experiment prescription) 2. Conscious or unconscious use (using journals and scientific websites, aware of the process). 3. Beliefs (belief or disbelief in necessity).

**Conclusion:**

It sounds essential to change the behavior of clinical faculty members from passive to active with respect to employing evidence-based medicine as well as to alter negative attitudes into positive ones. In so doing, systematic training program aiming at behavior changing is necessary. Also, providing dissent facilities and infrastructures and removing barriers to the use of EBM can be effective.

## 1. Introduction

Many clinical questions have no precise answers in performing daily medical activities. However, the patients expect the best treatment whereas doctors get their clinical treatment scenarios from past experiences (Khan, Dunning, Parvaiz, Agha, Rosin, & Mackway-Jones, 2011). Evidence-based medicine (EBM) is considered as a proper, wise method of using the best available evidence to take clinical decisions in treatment and care of patients (Sadeghi, Khanjani, & Moatamedi, 2011). Some have defined it as a judgment based on patient needs and scientific credible findings (Adib Hajbaghery & Azizi-Fini, 2012). Currently, EBM has been accepted as a reliable approach (Tomlin, Deamess, & Badenoch, 2002) which improves the patient-care process. Despite the fact that awareness and skills relevant to EBM are of significant factors in using this therapeutic method (Rangraz-jedi, Moraveji, & Abazari, 2013). Various studies suggest that clinicians know little of EBM (Prior, Wilkinson, & Neville, 2010). And due to lack of necessary skills in all conditions, evidence-based approach is not employed to provide clinical care services (Dawley, Bloch, Suplee, Mackeever, & Scherzer, 2010). Although awareness and the skills of using EBM are low (Raohani, Akbari, & Moradian, 2011), different studies show that half the treatments in the chosen hospital wards have not been based on scientific credible evidence (Ataei-Kachoei et al. 2010). Some earlier studies have argued that some 10 to 20% of treatments are in agreement with scientific valid evidence (White, 1995) and it needs to be highly generalized as scientific studies prove that when treatments are based on credible scientific evidence, the rate of patients’ recovery would be considerable (Davies et al., 1994, Mirzaei & Zahmatkesh, 2013, Rangraz-jedi, Moraveji, & Abazari, 2013). In addition to lack of awareness and adequate skills to use EBM (Khami et al., 2012, AdibHajbagheri, 2007), time limitation (Amada & Jones-Harris, 2003), lack of facilities and lack of proficiency in research skills, lack of information systems to access database and internet-based hygienic information are of some barriers to use this process (Khami et al., 2012). In order to overcome these problems, it is suggested we use an EBM short-term training intervention program (Rangraz-jedi, Moraveji & Abazari, 2013). Also, some believe that professional efficiency, practical knowledge, clinical experience, dissent environment of clinical education and having professional self-belief are of the most important factors in clinical decision- makings (Mohammadikonari et al., 2011). Yet, some state that evidence available in scientific sources are provided by researchers and are not the result of experiences of clinical experts. So, its implementation would face some problems at the practical stage (Grahame-Smith, 1995). What is currently considered as EBM is not practically performed in many cases (Nasr, 2010) because there aren’t sufficient and valid evidence for decision-taking, so clinicians practice more based on their own experience (Moeintaghavi, 2012; Madden, 2012). In addition to using experiences, reference books are the most important source for doctors in EBM (Sadeghi et al., 2011; Adib Hajbaghery, 2007) and a few use articles as well (Sadeghi et al., 2011). It seems that clinicians need to use available evidence adopted from high-level research findings performed by sophisticated individuals, merge them with subjective experiences and interpretations and finally implement them as a pragmatic therapeutic attempt. Studies suggest that some doctors don’t use EBM (Madden, 2012). When systematic information is provided, some take EBM as a process to promote the quality level of hospital’s clinical services and patients’ safety. They link clinical judgments to three factors: attitude, subjective criteria, and conscious control of behavior affected by efficiency and easy-to-use evidence, interpersonal impressions, individual creativities in using IT and self-efficacy (Yuang, Cheng, & Chieun, 2012). Although EBM is an important part of recent research efforts for reforming health-care system, it requires real-life situations of community and patient (Diamond, 2012). The present study attempts to clarify the concept of evidence-based medicine in educational and therapeutic experiences of clinical faculty members of Kermanshah University of Medical Sciences.

## 2. Materials and Methods

This study was carried out using qualitative method of phenomenology type. The population consists of 12 clinical faculty members of Kermanshah University of Medical Science. Given the method of the study, sampling was done purposefully. In other words, for data gathering, we referred to individuals with clinical experiences of evidence-based medicine. The volume of sampling was determined using samples’ adequacy law. Here, data gathering continued to grow to saturation of expressed statements by clinical faculty members when the responses were repeated and there was no new case within, data gathering ended.

Data gathering was performed using semi-structured interview. General questions of the interview were predetermined. But since questions were open-ended, they were formed by the participants. Sample interview questions started with a general question of the interview: “would you deal with clinical work using EBM approach?” in case of any ambiguities or further explanations in the questions, it was asked the other way. Sample question: when doing clinical work or practice medicine, you encounter a new case you cannot diagnose using your background knowledge, whet ways would you take for clinical judgment?” followed by some other questions such as “what websites or database would you use?” and/or “how much would you consider this process to be useful?” “What process would you take for EBM decisions? Please explain more.” “Discuss the advantages and disadvantages of this process.” “Define the examples and samples which have helped you get to clinical diagnosis based upon scientific evidence and findings.”

The interviews were carried out by appointment at the participants’ workplace and each interview took about 15 to 30 min. the interview texts were both noted and recorded simultaneously. The recorded interview was transcript right away and adopted with the notes. Then, the components were coded. Two individuals were used for coding, agreement coefficient between these individuals’ codes was calculated 95% and considered as data reliability. To be consistent with the ethics of research, vice president of Research and Technology of University communicated with the participants. The individuals took part voluntarily in the project and in the case of willingness they continued the interview, otherwise they excluded. In dissemination of information, confidentiality and non-disclosure of the names were respected.

To interpret the data, inferential-interpretive approach was used meaning that the coded cases were turned into sub-categories with reduction method and then the sub-categories were merged together and formed the main categories. Finally, data interpretation was done using Collaizzi pattern (Abedini et al., 2011) concurrent with data gathering. Interpretation procedure was a follows: 1. Participants’ explanations were transcribed and read several times. 2. Important statements relevant to the phenomenon under examination were determined and written. 3. Important relevant statements were coded. 4. The coded statements were clustered and organized in main topic and (themes). 5. A general description of extracted main topics was provided. 6. An overview of the phenomenon’s natural structure (evidence-based medicine) was provided.

## 3. Results

Characteristics of participants: a total of 12 experts of faculty members of Kermanshah University of Medical Sciences doing clinical work currently participated in this study. These people have specialties in internal medicine, gastroenterology, cardiology, pediatrics, neurosurgery, general surgery and pathology.

The main themes and sub-categories: interpreting the data, three main themes were extracted: 1. Unawareness and lack of using EBM 2. Conscious and unconscious use of EBM 3. Beliefs on EBM were extracted. Each main theme had sub-categories. For main theme of “unawareness and disuse of EBM” there were four sub-categories: 1.1 unaware of EBM 1.2 disuse of this process 1.3 referral to clinical colleagues 1.4 there were prescriptions of tests and para-clinical attempts. For the main theme conscious and unconscious use of EBM, there were three sub-categories: 2.1 using journals 2.2 using scientific web-sites 2.3 awareness of EBM was extracted. Also, for the main theme of attitudes on EBM, there were two components: 3.1 beliefs on the necessity of EBM and 3.2 disbeliefs on the necessity of EBM were obtained. Findings are presented briefly in Table 1.

**Table 1:** Presentation of main themes, sub-categories and sample of components by clinical faculty members of Kermanshah University of Medical Sciences

Major Categories	Sub-categories	Explanations and Sample Comments
Unaware and Disuse of EBM	Unaware of Concept	sample of comments: I haven’t heard of it, I almost know nothing of it, I have no idea and haven’t heard of it yet, I haven’t heard of this phrase so far, I haven’t trained in this area.

	Disuse of EBM	Other people don’t use it. I have no idea so I don’t use it and it isn’t used. It is used slightly. It is still a long way for our country to know that this method is useful. Doctors who prescribe the same for all patients and consider the patients similarly don’t use this method. If the adults do it, they inspire the minors…

	Referral to Clinicians	We send them to another doctor who specializes in this field and say that this is my diagnosis and seek his idea. I send to a doctor who is specialized or more experienced.

	Prescription of Test and Para-clinical Efforts	If it is a chronic disease and we cannot diagnose, we prescribe experiments and so on. Unless I get them to do some diagnostic processes and wait to get ultrasound and C.T. results, I do search the same day.

Conscious or Unconscious Use of EBM	Using Journals	At the first place, I use journals.

	Using Scientific Websites	If I get nowhere through consultancy and colleagues, I will search through websites- first, I go to books, then the colleagues and finally the websites: scientific ones such as Google Scholar. I’d rather using websites to my earlier hand-written notes. If there is an ambiguous case, I do search and get some articles from credible websites and journals and examine the solutions. Then, I follow the treatment process. I use Up to Date Medicine and Medscape….

	Having Info & Awareness on EBM	I became familiar with this phrase when I was in Isfahan, when I was a university student. Back then I took part in its workshops. As we are faculty members and committed and we hold some sessions per month and are quite familiar with this phrase and we use it in our processes but the rest of people don’t have to. I am quite familiar with EBM and use it. I regard it as doctors’ consultation in rare cases along with various searches to get to a comprehensive idea.

	Consultancy with colleagues	I consult or call my colleagues. I consult with pathologist colleagues like dr. … or with specialized ones. If searching is pointless, I do consult with others and ask other professors, experienced or in experienced, if they have ever encountered this problem or not. At my residency, there was a patient with meningitis with no natural trend. Having consulted the professors, he was diagnosed with lupus. First of all, I consult with professors, then my own personal searches. I search the websites if consultancy with colleagues is pointless…

Beliefs on EBM	Using EBM method	I put off the treatment for searching information. I use this method in my treatment process. In fact, I search for newer things and why it has lost its natural process? Then, I notice that I should search. I searched based on the case I had. After patient’s examination, I do search the same day since I should take my decisions rapidly. I am always connected and search the unknown case right away. I spend the treatment process based on my findings. I search 3 to 4 articles per night for side effects of a drug or illness symptom. I search the latest developments. For example, a patient takes drugs which may have interposing side-effects. I do my searches and tell him the results tomorrow. For instance, the patient is diagnosed with psoriasis or fat liver. Then, he asks me about the relationship. If I am informed, I’ll answer. If not, I ask them to come back for the results later and I do my searches during this time since I have little information on drugs (I am specialized in another field.)

	Belief on Importance and Necessity of EBM	Using this method would help in diagnostic and para-clinical efforts. Diagnosis and treatment trend happen fast. Tumor cases are large. There is a new case everyday coming up and we should comment on it. Some time ago, a baby was born with two short and two long members. And there is no similar case mentioned in any books. Using the websites in these cases is significant. If Health and Treatment Ministry stresses that no one patients would ever face any problems with this method, if diagnosis and treatment happen based upon the evidence, it bears lots of benefits for the patients. When the books are helpless, using websites is essential (EBM). I believe this is very efficient but it requires pre-training. There are many new cases of internal patients and the search is essential. I put off the treatment process and draw conclusions after the search: one of the benefits is that if there is a patient with gastrointestinal hemorrhage, all the judges would be the same. But, they search; they realize that it may be of another type….

	Belief on Non-necessity of EBM	I go to several hospitals. I get home at 9. I do the chores and I have no time for studying or research. I should make money and earn a living. A pediatrician examines a patient of 99 just for making money. EBM doesn’t work for us. In emergency cases, there is no opportunity to visit. It isn’t used because it isn’t realistic. As there isn’t sufficient internet in hospital, it is slightly used (just in library). There is a long way a head in our country to believe that this is a useful method. If you search for something, they may say you are not updated. When you search or read a book, the patient may lose its faith in you. Doctors, who prescribe the same drugs for all patients and see the patients alike, don’t use this method. If the adults or more precedent do this, they will be inspiring for the minors.

## 4. Discussion

The findings suggested that some participants were not familiar with the concept of evidence-based medicine scientifically, but they used this method in their own experiences. To confirm the recent study’s findings, various studies suggested that clinicians know little of EBM (Sadeghi et al., 2011; Raohani, Akbari & Moradian, 2011; Adib Hajbaghery, 2007) and haven’t got the essential skills to use this method (Dawley et al., 2010). Also, their awareness and skills of EBM were low but half the treatments done in the wards of the chosen training hospitals were based on scientific credible evidence (Ataei-Kachoei et al., 2010). Earlier studies showed that some 10 to 20% of the treatments are in agreement with credible scientific evidence (White, 1995). The results of a study suggested that 80% of dental students had a little or little knowledge of evidence-based dentistry (Khami, 2012) whereas awareness and skills relevant to EBM are of important factors of using this treatment (Prior, Wilkinson, & Neville, 2010). The studies in this regard give reasons for justifying major factors of unawareness on this issue scientifically. Some has considered time limitation (Amada & Jones-Harris, 2003) and some has taken the lack of useful educational interactions to develop EBM skills, decision-taking skills and clinical judgments as the source of unawareness (Rangraz-jedi, Moraveji, Abazari, 2013; Rangraz-jedi, Moraveji, & Abazari, 2013; Eliot, 2004). Given that scientific studies confirm this issue that treatments based on scientific credible evidence are effective in the improvement of clinical attempts (Mirzaei, & Zahmatkesh, 2013; Davies, 1994, Yuang hung et. al., 2012) and currently it is accepted as a reliable approach (Tomlin, Deamess, & Badenoch, 2002) and this is applied when awareness level is high (Prior, Wilkinson, & Neville, 2010; Dawley et al., 2010; Moeintaghavi et al., 2014), it sounds essential to raise awareness of clinical faculty members in this regard. The findings showed that the patients don’t use EBM in some clinical attempts and the reason is to exploit alternative decisions such as: prescription of Para-clinical efforts, referral to other specialists, using self-experiences and cases like: lack of awareness, lack of facilities and infrastructures, and disbelief to the efficiency of this method. Studies showed that doctors practice their clinical treatment based on their past clinical experiences (Nasr, 2010; Moeintaghavi et al., 2012). Some would take it as the setback of creativity and innovation in their own treatment (Madden, 2012). One study indicated that lack of facilities, time and proficiency to research skills are the most obstacles of evidence-based performance (Adib Hajbaghery, 2007). It has also been stated that Iran’s hospital information systems lack sufficient parts to access support system of clinical decision, reference database, and internet-based hygienic information (Khami, 2012). Some also state that available scientific evidence in scientific sources has been prepared by researchers. Thus, its implementation would face some problems (Grahame-Smith, 1995), though it isn’t an appropriate justification not to use this process because university faculty members have equal facilities for their own clinical attempts and the results suggested that the participant would use this process.

The findings showed that from the viewpoint of experiences of clinical faculty members of university “EBM” equals using books, scientific journals, consulting with colleagues, referral to hand-written notes of academic years and surfing scientific websites. EBM is defined as a wise and correct way of using the best available evidence for clinical decisions in treating and caring the patients (Sadeghi et al., 2011) and also judgment based on the patient’s need and credible scientific findings (Adib Hajbaghery & Azizi-Fini, 2012). According to research findings, due to lack of sufficient and valid evidence, professors would practice mostly based on their experiences (Nasr, 2010). It seems that clinicians need the evidence adopted from high-level research findings carried out by sophisticated individuals practically to take decisions, which are merged with subjective experiences and interpretations and finally put forward as a pragmatic treatment (Madden, 2012). In the meantime, reference books, researches-experiences and individuals’ background information are true evidence so much so that the main source of information for clinical decision-taking has been defined to be books, clinical experiences with books and articles (Sadeghi et al., 2011). According to results of the present study and other researches in this field, it seems that EBM is not a single version prescribed for all clinical decision-taking situations similarly. Rather, based upon past situations and experiences, the specialists would use it with respect to facilities and time. However, participants stress more on background and colleague knowledge so as to find the best evidence using new scientific findings. Moreover, instead of routine and permeantuse, clinicians would use this process when they face a new case in which they fail to find any evidence in their experiences for judgment and taking decisions. These examples show the salient difference between EBM stated in credible valid sources and what is practically used by clinicians.

Based upon the findings of this study, one of the main themes was “clinicians’ beliefs to EBM”. This included the sub-component of “belief in the importance and necessity of EBM.” Similarly, Rangrajedie et al., (2013), showed that doctors had consensus on EBM and they all found it helpful. Khami and et al., (2012), gained these findings in their study that 80% of the participants were in agreement with this approach (Khami et al., 2013). Moeintaghavi et al., (2014), in their study under “examining the rate of awareness and using evidence-based dentistry” among dentists realized that 96% of dentists argued that using evidence- based dentistry for taking care of the patients would have better clinical results. The next component was “belief in non-necessity of EBM” gained from experiences of clinical faculty members of university. Although EBM is based on attitude, faculty members don’t hold a positive attitude to this process for various reasons (Yuang Hung, Cheng, & Chi Chieun, 2012). They think the major reason is various obstacles on the way of EBM. In this regard, some state that available scientific evidence would face some problems practically (Grahame-Smith, 1995). Maden (2012) assessed EBM as an obstacle on the way of clinical creativity in practice. Rangrezjedi et al. (2013), drew this conclusion that the necessity of training new skills is an obstacle to EBM. Lack of facilities, time and proficiency in research skills are of the most important obstacles of EBM performance, affecting the level of awareness, attitude and EBM performance (Adib Hajbaghery, 2007).

## 5. Conclusion

In general, three main themes were extracted. They included: unawareness and disuse of EBM, conscious and unconscious use of EBM, and beliefs on EBM. Each main theme had sub-categories. For the main theme of “unawareness and disuse of EBM”, there were four sub-categories: unaware of the concept of EBM, disuse of this process, referral to clinical colleagues, prescription of tests and para-clinical efforts. For the main theme of conscious and unconscious use of EBM, there were three sub-categories: using journals, scientific websites, and unaware of EBM process. Also, as for the main theme of beliefs on EBM, there were two sub-categories: belief and disbelief on the necessity of EBM. It sounds essential to change the behavior of clinical faculty members from passive to active regarding EBM and to change negative attitudes to positive ones. In this respect, systematic training planning is required for changing the behavior. Moreover, providing dissent facilities and infrastructures and removing the obstacles on the way of exploitation of EBM can be effective.
